# Neoadjuvant Chemotherapy-Induced Downstaging Enables R0 Craniofacial Combined Resection in AJCC (American Joint Committee on Cancer) Stage IV Olfactory Neuroblastoma: A Case Report of 12-Year Disease-Free Survival

**DOI:** 10.7759/cureus.100226

**Published:** 2025-12-27

**Authors:** Akira Nakazono

**Affiliations:** 1 Otolaryngology-Head and Neck Surgery, Hokkaido University, Sapporo, JPN

**Keywords:** craniofacial resection, esthesioneuroblastoma, neoadjuvant chemotherapy, olfactory neuroblastoma, skull base tumor

## Abstract

Olfactory neuroblastoma (ONB), also known as esthesioneuroblastoma, is a rare malignant tumor arising from the olfactory neuroepithelium, and optimal management of advanced cases with extensive intracranial extension remains challenging. Neoadjuvant chemotherapy (NAC) is not routinely recommended in current guidelines but may be considered in selected patients with locally advanced or initially unresectable disease. We report the case of a 36-year-old man who presented with recurrent epistaxis and progressive somnolence. Nasal endoscopy, computed tomography, and magnetic resonance imaging revealed a large mass occupying the nasal cavity and paranasal sinuses with massive extension into the anterior cranial fossa. Endoscopic biopsy confirmed ONB, staged as modified Kadish C, Dulguerov T4N0, according to the American Joint Committee on Cancer (AJCC) 8th edition, as cT4cN0cM0, clinical Stage IV. Following multidisciplinary tumor board review, curative resection as an initial treatment was considered unfeasible due to the marked intracranial extension, NAC with ifosfamide, cisplatin, and etoposide (ICE) was initiated. During NAC, the patient developed grade 3 neutropenia, grade 3 anemia, and grade 4 thrombocytopenia according to Common Terminology Criteria for Adverse Events (CTCAE) version 5.0, requiring granulocyte colony-stimulating factor support and platelet transfusion; however, these toxicities were successfully managed, and five cycles of ICE were completed without delaying surgery. Follow-up imaging demonstrated marked tumor reduction with a substantial decrease of the intracranial component, corresponding to a partial response. Subsequent endoscope-assisted craniofacial resection achieved gross total tumor removal, and histopathological examination of the resected specimen confirmed ONB. Adjuvant three-dimensional conformal radiotherapy with a total dose of 60 Gy in 30 fractions was administered. At 12 years and six months after surgery, the patient remains alive and disease-free. This case suggests that, in carefully selected patients with advanced ONB, NAC can achieve meaningful tumor downstaging and enable curative surgery with durable long-term disease control.

## Introduction

Olfactory neuroblastoma (ONB), also known as esthesioneuroblastoma, is a rare malignant tumor originating from the olfactory neuroepithelium. Since its first description by Berger et al. in 1924 [1], numerous cases have been reported, and cervical lymph node metastases have been observed in approximately 10-15% of patients, whereas distant metastases occur in fewer than 10% [2,3]. Standard treatment consists of complete surgical resection followed by postoperative radiotherapy [4,5], although neoadjuvant chemotherapy (NAC) may be considered in cases where upfront surgery is technically difficult or anticipated to be highly morbid. However, management of advanced ONB with extensive intracranial extension remains challenging, as complete resection is often considered unfeasible. Here, we report a case of advanced ONB with massive intracranial invasion that was successfully treated with NAC consisting of ifosfamide, cisplatin, and etoposide (ICE), which resulted in significant tumor downstaging and enabled complete craniofacial resection, ultimately achieving long-term disease-free survival (DFS) of 12 years and six months.

## Case presentation

**Figure 1:**
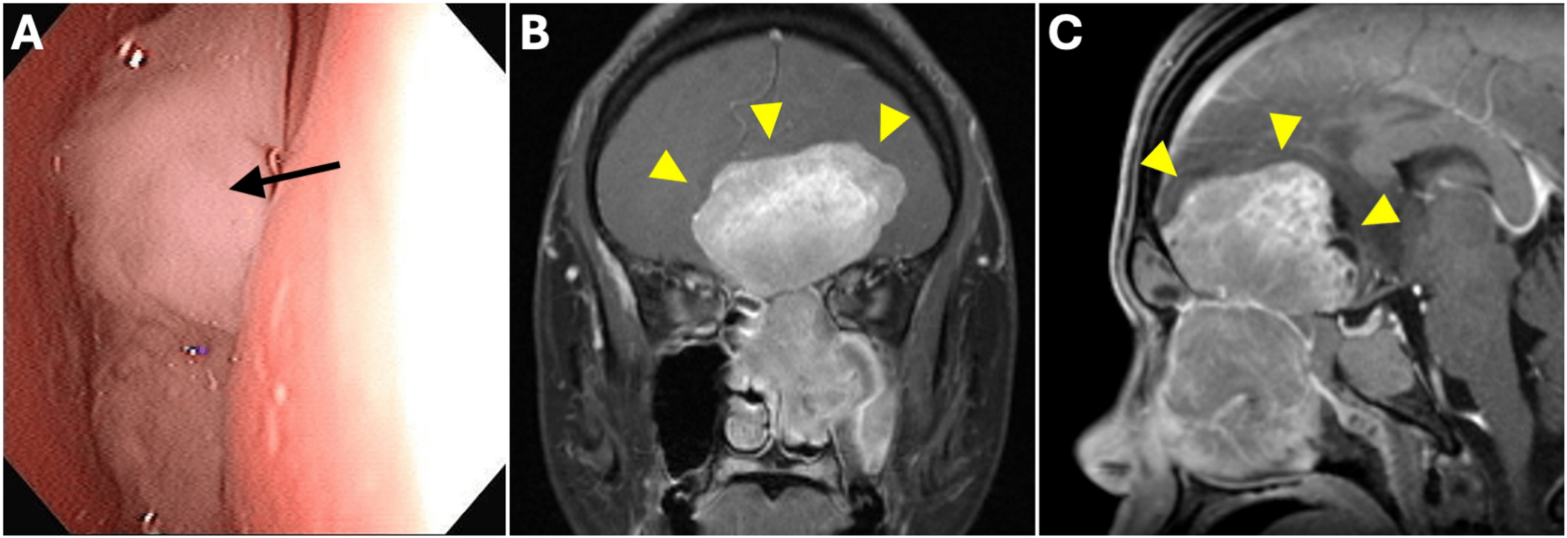
Initial findings at the presentation. (A) Nasal endoscopy showing a whitish tumor completely filling the left nasal cavity (black arrow). (B, C) Gadolinium-enhanced T1-weighted magnetic resonance imaging revealed a well-defined space-occupying lesion in the left olfactory groove, measuring approximately 8.2 cm × 4.8 cm × 5.6 cm, with a characteristic dumbbell configuration (yellow arrowhead). The lesion extends inferiorly into the left ethmoid, frontal, and maxillary sinuses, and superiorly into the intracranial compartment, involving the inferior aspect of the left frontal lobe and the olfactory tract. It causes significant mass effect with contralateral displacement of the frontal midline structures by 25°. On contrast enhancement, the lesion demonstrates heterogeneous signal intensity. Notably, the distance between the posterior tumor margin and the supraclinoid segment of the left internal carotid artery exceeds 1 cm, indicating no direct involvement of this critical vascular structure.

A 36-year-old male patient with no history of smoking or alcohol consumption presented to the otolaryngology department of our hospital with recurrent epistaxis and progressive somnolence. He had no significant past medical history and was previously healthy, with no known chronic conditions. His occupation was that of an office worker. At the initial visit, nasal endoscopy, CT, and MRI were performed. Nasal endoscopy revealed a large mass occupying the nasal cavity. CT and gadolinium-enhanced MRI demonstrated a huge, well-circumscribed tumor measuring approximately 8.2 × 4.8 × 5.6 cm, centered in the left nasal cavity and paranasal sinuses, with extension into the anterior cranial fossa and marked intracranial invasion. The lesion exhibited a dumbbell configuration and caused significant mass effect with contralateral displacement of the frontal midline structures. Despite the extensive intracranial extension, no direct involvement of the supraclinoid segment of the left internal carotid artery was observed (Figures 1A-1C). Endoscopic biopsy of the nasal cavity mass led to a diagnosis of ONB, Hyams grade II [6]. There was no evidence of cervical lymph node or distant metastasis, and the disease was staged as modified Kadish C [7], Dulguerov T4N0 [8] and according to the American Joint Committee on Cancer (AJCC) 8th edition [9], as cT4cN0cM0, clinical Stage IV.

**Figure 2:**
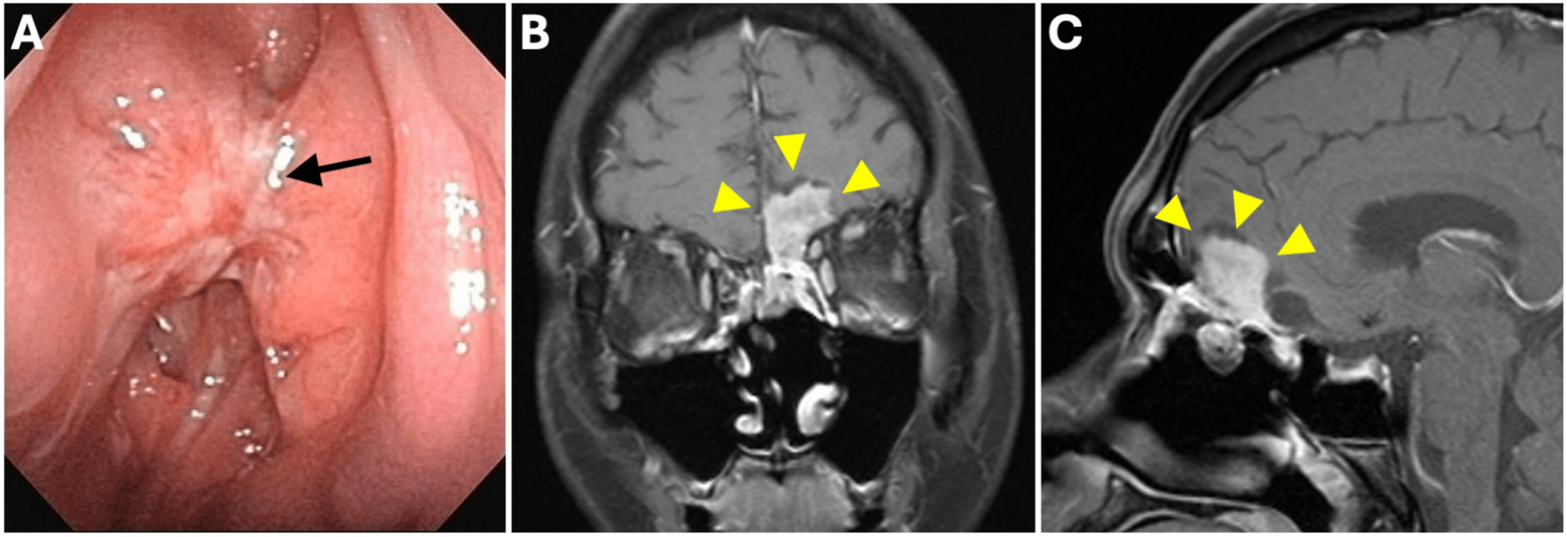
Findings after five cycles of neoadjuvant chemotherapy. (A) Nasal endoscopy confirmed a reduction in the size of the nasal cavity mass (black arrow). (B, C) Gadolinium-enhanced T1-weighted magnetic resonance imaging revealed marked reduction in the size of the lesions located in the nasal cavity, paranasal sinuses, and intracranial compartment, now measuring approximately 3.2 cm × 1.2 cm × 2.5 cm, with a persistent dumbbell configuration (yellow arrowhead). The lesion components within the left maxillary and frontal sinuses have nearly resolved, while those in the left ethmoid sinus and inferior aspect of the left frontal lobe show significant regression. The midline structures of the frontal lobe have reverted to their normal anatomical position. Post-contrast imaging demonstrates heterogeneous enhancement within the residual lesion.

Following careful discussion at a multidisciplinary tumor board involving otolaryngology, head and neck surgery, radiation oncology, neurosurgery, and medical oncology, the marked intracranial extension was considered likely to result in residual disease if upfront surgery were performed. Therefore, given the extent of disease and the anticipated morbidity of initial resection, NAC with the ICE regimen (ifosfamide 900 mg/m², cisplatin 20 mg/m², and etoposide 60 mg/m²) was initiated. During NAC, according to the Common Terminology Criteria for Adverse Events (CTCAE) version 5.0 [10], the patient developed grade 3 neutropenia, grade 3 anemia, and grade 4 thrombocytopenia, requiring administration of granulocyte colony-stimulating factor (G-CSF) and platelet transfusion. Because a clear antitumor effect of ICE was observed during treatment, NAC was cautiously continued and completed for a total of five cycles (cumulative doses: ifosfamide, 4,500 mg/m²; cisplatin, 100 mg/m²; etoposide, 300 mg/m²). After completion of NAC, follow-up CT and MRI demonstrated marked tumor reduction, with the lesion decreasing in size from approximately 8.2 × 4.8 × 5.6 cm before treatment to 3.2 × 1.2 × 2.5 cm after treatment, particularly reflecting a substantial reduction of the intracranial component. Tumor volume, estimated using the ellipsoid approximation method (π/6 × length × width × height), showed an approximate volume reduction of 95%, corresponding to a partial response according to the Response Evaluation Criteria in Solid Tumors (RECIST), version 1.1 (Figures 2A, 2B) [11]. Five months after the initial visit, combined endoscope-assisted craniofacial resection was performed, achieving gross total removal of the tumor.

**Figure 3:**
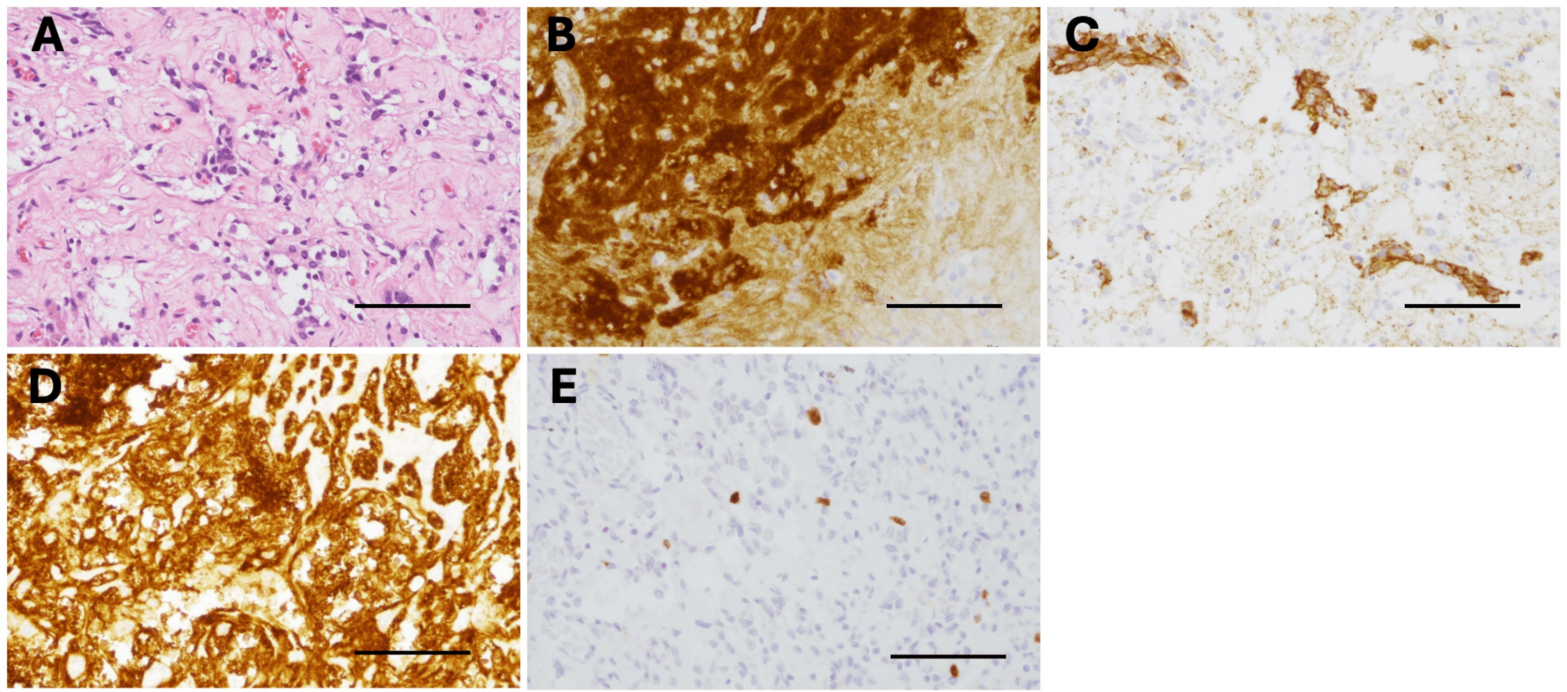
Histopathological findings of the tumor. (A) Hematoxylin and eosin staining showing tumor cells with clear cytoplasm and enlarged nuclei proliferating in small clusters or gland-like structures. (B) Immunohistochemistry for synaptophysin shows positive staining in the tumor cells. (C) Immunohistochemistry for chromogranin A shows positive staining in the tumor cells. (D) Immunohistochemistry for CD56 shows positive staining in tumor cells. (E) The Ki-67 labeling index was less than 5%. All scale bars indicate 100 µm.

Histopathological evaluation of the resected specimen revealed gliosis, fibroblast proliferation, and capillary hyperplasia. Tumor cells exhibited clear cytoplasm and enlarged nuclei, forming small clusters or glandular structures. Immunohistochemical staining was positive for synaptophysin, chromogranin A and CD56, confirming the diagnosis of ONB (Figures 3A-3D). The Ki-67 labeling index was less than 5% (Figure 3E).

**Figure 4:**
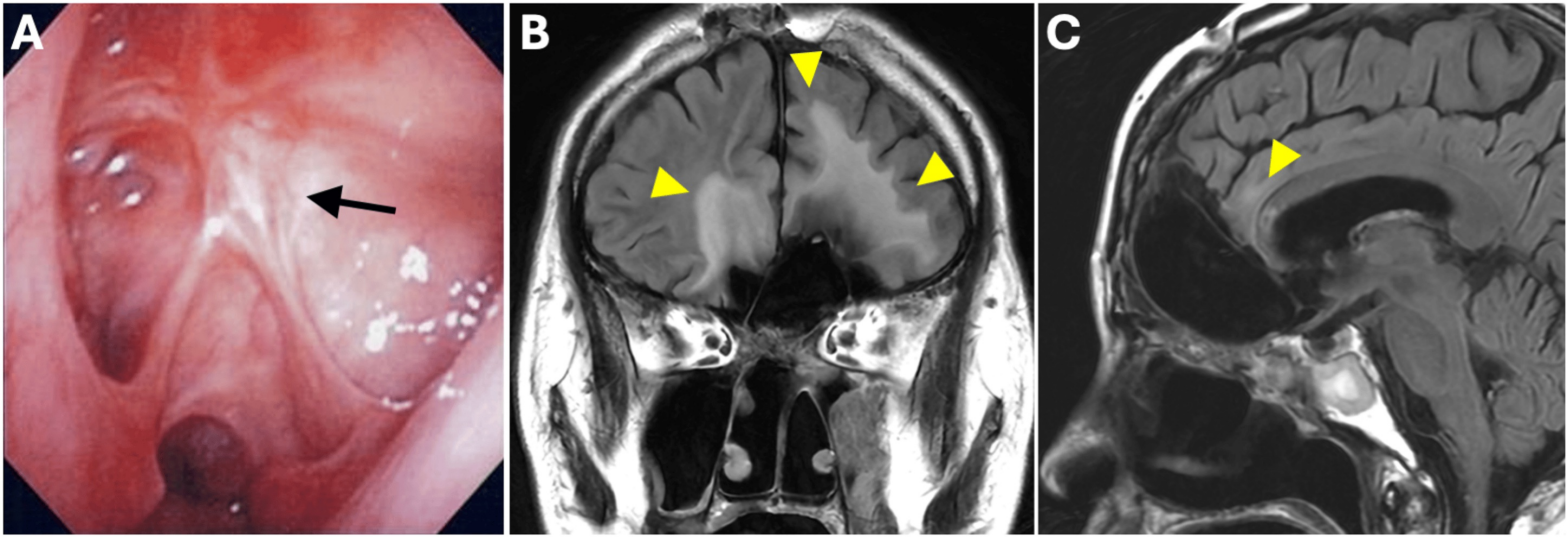
Findings 12 years after the surgery. (A) Nasal endoscopy revealed no evidence of local recurrence in the nasal cavity (black arrow). (B, C) Gadolinium-enhanced T1-weighted magnetic resonance imaging revealed edematous changes consistent with prior radiotherapy (yellow arrowhead) but no obvious evidence of tumor recurrence.

The postoperative course was uneventful. The patient received adjuvant radiotherapy with a total dose of 60 Gy in 30 fractions using three-dimensional conformal radiotherapy (3D-CRT). At 12 years and six months after surgery, the patient remains alive and disease-free (Figures 4A-4C).

## Discussion

NAC for ONB remains a controversial topic. Although current treatment guidelines do not recommend NAC as a routine component of ONB management, several reports have suggested that it may be beneficial in selected patients. In particular, chemotherapy has been reported to be effective in patients with locally advanced disease [12], as well as in those with Hyams grade III/IV or Kadish group C/D tumors [13,14]. Conversely, other studies have indicated that the therapeutic benefit of chemotherapy is limited in ONB [15,16], emphasizing that ONB generally remains a surgically driven disease. Early surgical intervention is considered crucial [17], and in a large series evaluating 797 patients, induction chemotherapy was associated with worse outcomes, presumably due to delays in definitive surgical treatment [18]. Taken together, these findings highlight that while NAC may have a role in highly selected settings, its indiscriminate use carries potential risks and should be approached with caution.

In the present case, the primary lesion exhibited massive intracranial extension and was initially considered unresectable or resectable only at the cost of unacceptable neurological morbidity. Against this backdrop, where NAC for ONB is controversial and where some reports even suggest harm from delaying surgery [18], the decision to administer ICE chemotherapy was made with the specific goal of tumor downstaging and reassessing resectability. The marked tumor regression achieved after completion of five cautiously administered cycles of ICE converted an essentially inoperable lesion into one amenable to gross total resection by combined craniofacial surgery. Importantly, this radiological response translated into a clear surgical advantage, allowing an oncologically sound resection while avoiding excessive manipulation of the frontal lobes and critical neurovascular structures. The subsequent long-term DFS of more than 12 years suggests that, in carefully selected patients with extensive skull base and intracranial involvement, NAC can contribute to curative treatment despite the concerns raised in prior large-cohort analyses.

The ICE regimen was selected based on its established activity in neuroectodermal and other small round blue cell tumors. Mechanistically, the combination of ifosfamide, cisplatin, and etoposide delivers complementary DNA-damaging effects (alkylation, platinum-induced crosslinking, and topoisomerase II inhibition), an approach that has shown clinical activity in other neuroectodermal malignancies and may plausibly account for the marked tumor regression observed in our ONB case [19]. In line with this rationale, Kim et al. reported objective responses in 9 of 11 patients (objective response rate, 82%, including two complete responses and seven partial responses) treated with neoadjuvant etoposide, ifosfamide, and cisplatin, with a median survival of 18 months (range: 3-45 months) [19]. However, ONB-specific evidence supporting ICE remains limited. Nevertheless, several prior reports have described meaningful responses to NAC in locally advanced or initially unresectable ONB, supporting its selective use when downstaging is needed to facilitate complete resection [12-14]. In the present patient, grade 3/4 hematologic toxicities, including neutropenia, anemia, and thrombocytopenia, were observed during NAC; however, these adverse events were appropriately managed with granulocyte colony-stimulating factor support and platelet transfusion, allowing completion of the planned five cycles without delaying the scheduled surgical treatment. This point is particularly relevant given the concerns that NAC may postpone definitive surgery and thereby adversely affect oncologic outcomes [18]. Our experience suggests that, under close monitoring and with timely supportive care, even intensive regimens such as ICE can be delivered safely enough to preserve the intended treatment timeline.

The role of radiotherapy also deserves mention. Postoperative radiotherapy is generally considered a key component of multimodal therapy for ONB, particularly in cases with advanced local disease [20]. In our patient, adjuvant 3D-CRT with a total dose of 60 Gy in 30 fractions was administered to the skull base region, in line with commonly used dose ranges. Long-term follow-up MRI demonstrated only edematous changes compatible with prior irradiation and no evidence of local recurrence, supporting the durability of local control achieved by the combination of NAC, surgery, and adjuvant radiotherapy. The absence of recurrence 12 years after treatment provides reassuring evidence that this multimodal strategy can yield excellent long-term oncologic outcomes in selected patients.

Several limitations of this report must be acknowledged. First, this is a single case, and the dramatic response to ICE chemotherapy observed here cannot be generalized to all patients with ONB. Chemosensitivity likely varies according to tumor biology, including Hyams grade, Kadish stage, and underlying molecular alterations. Second, robust prospective data on NAC in ONB are lacking, and the existing literature is largely composed of retrospective series and case reports with heterogeneous treatment protocols, response criteria, and follow-up durations. Third, the optimal number of NAC cycles and the most appropriate radiologic and pathologic endpoints remain undefined. Given that early surgical intervention has been associated with improved outcomes and induction chemotherapy may negatively impact prognosis by delaying definitive surgery in some patients, the decision to employ NAC should be made only after thorough multidisciplinary discussion, taking into account the extent of disease, expected resectability, patient performance status, and institutional experience with skull base surgery and intensive chemotherapy.

Future research should focus on clarifying the precise role of NAC within the overall treatment algorithm for ONB. Multicenter collaborative studies and registry-based analyses are needed to better define response rates, survival outcomes, and toxicity profiles associated with various neoadjuvant regimens, including ICE-based combinations. Furthermore, comprehensive histopathological and molecular profiling, including immunohistochemistry, genomic sequencing, and transcriptomic analysis, should be performed with a focus on well-established driver genes in ONB, such as MYC and TP53, as well as key chemotherapy-related pathways, particularly the DNA damage repair pathway, to identify predictive biomarkers of chemotherapeutic response. This strategy enables more precise patient selection for NAC and helps avoid unnecessary treatment-related toxicity and surgical delays in patients who are unlikely to respond. To reduce the risk of adverse effects and prevent postponement of surgery, predefined mid-treatment efficacy evaluation points, such as imaging reassessment after two cycles of the ICE regimen, should be established, with immediate discontinuation of NAC and prompt transition to surgical intervention in patients exhibiting disease progression or lack of response. Additionally, a maximum number of chemotherapy cycles or a fixed treatment duration (e.g., no more than 12 weeks) should be prospectively defined to prevent overtreatment and ensure timely access to surgery within the optimal therapeutic window. As additional evidence accumulates, it may become possible to reconcile the conflicting data in the literature and to establish clearer indications, such as specific staging, grading, or molecular subgroups, for neoadjuvant therapy in patients with locally advanced or initially unresectable ONB.

## Conclusions

Neoadjuvant ICE chemotherapy may represent a valuable therapeutic strategy for carefully selected patients with advanced ONB initially considered unresectable, particularly those presenting with locally advanced or intracranial extension, as exemplified by the AJCC stage IV case described in this report. In this patient, chemotherapy-induced tumor regression was substantial, resulting in disease downstaging and enabling curative-intent R0 craniofacial resection, a critical determinant of long-term survival, which ultimately led to 12 years and six months of postoperative DFS. Nevertheless, due to the inherent heterogeneity of ONB and the current absence of standardized guidelines for neoadjuvant treatment, further prospective, multicenter studies with larger patient cohorts are warranted to establish: (i) precise clinical and pathological criteria for patient selection; (ii) reliable biomarkers predictive of response to neoadjuvant ICE therapy; and (iii) the optimal sequencing of chemotherapy and surgery to maximize oncologic efficacy while minimizing treatment-related morbidity.
